# Implementing a Social Accountability Approach for Maternal, Neonatal, and Child Health Service Performances in Ethiopia: A Pre-Post Study Design

**DOI:** 10.9745/GHSP-D-20-00114

**Published:** 2021-03-31

**Authors:** Mesele D. Argaw, Binyam Fekadu Desta, Elias Mamo, Melkamu G. Abebe, Deirdre Rogers, Anteneh Demelash, Aklilu A. Ayele, Zinabu Reda, Amare S. Tareke, Alemu M. Erfo, Wegayehu W. Wonjalo, Temesgen A. Bele, Assefa Ayede, Lidya G. Abebe

**Affiliations:** a U.S. Agency for International Development Transform: Primary Health Care Activity, JSI Research & Training Institute, Inc., Addis Ababa, Ethiopia.; b JSI Research & Training Institute, Inc., Boston, MA, USA.; c South Wollo Zone Health Department, Dessie, Ethiopia.; d Kembata Tembaro Zone Health Department, Durame, Ethiopia.; e Ethiopian Federal Ministry of Health, Health Extension Program and Primary Health Care Directorate, Addis Ababa, Ethiopia.; f Ethiopian Federal Ministry of Health, Reform and Good Governance Directorate, Addis Ababa, Ethiopia.; g Addis Ababa University, Schools of Public Health, Addis Ababa, Ethiopia.

## Abstract

**Background:**

Social accountability approaches, such as the community scorecard (CSC), can improve the performance of health systems in low-income countries by providing a mechanism for obtaining and incorporating community input. This longitudinal study assessed the effects of CSCs implemented by primary health care units (PHCUs) on health system performance in Ethiopia.

**Methods:**

This study used a pre-post design and was conducted from October 2018 to September 2019 in 159 PHCUs in 31 districts in Amhara and Southern, Nations, Nationalities and Peoples' regional states. The data were extracted from a routine health information management system database at baseline, midterm, and endline stages over 12 months for statistical analysis. The effects of implementing CSCs on health system performance were evaluated against selected key performance indicators (KPIs).

**Results:**

The CSC measurement results were based on input from 38,556 community representatives. The mean CSC score with standard deviation (±SD) was 60.8%±12.5%, 66.3%±10.8%, and 70.6%±10.0% at baseline, midterm, and endline, respectively. The mean KPI score was 54.9%±17.4%, 61.9%±15.1%, and 67.6%±14.6% at baseline, midterm, and endline, respectively. The average CSC and KPI values were positively correlated (r>0.37). Using a nonparametric Friedman's test, we found a statistically significant difference in CSC and KPI scores at baseline, midterm, and endline (*P*=.001). Post hoc analysis with Wilcoxon signed-rank tests was conducted with a Bonferroni correction and the results showed higher CSC and KPI values from baseline to midterm and from midterm to endline (*P*<.017).

**Conclusions:**

The use of CSCs in Ethiopia contributed to the health system's performance in terms of maternal and child health services. The responsiveness of health workers and utilization of basic health services by community members were found to increase significantly with CSC use. We recommend continued implementation of the CSC intervention at PHCUs.

## Abstract (precis)

Implementing a community scorecard approach may help increase utilization of maternal, neonatal, and child health services in primary health care facilities. The results of our study show the importance of engaging both the community and health workers to measure and continuously improve health care processes and improve the health system performance.

## Teaser

**Key Findings:**

- The average community scorecard measurements on health workforce behavior toward patients, availability of services, patient waiting time, facility infrastructure, ambulance service, and cleanliness and safety of the health facility significantly improved over 1 year at primary health care units.
- Of the 10 key maternal neonatal and child health performance indicators measured, 9 were found to improve as a result of implementing the community scorecard approach.

**Key Implications:**

- The implementation of a community scorecard approach enhances a culture of social accountability, transparency, and engagement of citizens in planning, implementing, and evaluating maternal, neonatal, and child health services. In addition, it improves the negotiation capacities and involvement of both community members and health workers, resulting in increased availability and utilization of health service.
- Program managers and development partners should continue their support for the government- led social accountability interventions to ensure the sustainability of improvements in maternal, neonatal, and child health outcomes.

## BACKGROUND

The World Development Report of 2004 highlighted the benefits of listening to citizens to improve pro-poor targeted service delivery.^1^ Following this report, the global community showed an eagerness to institutionalize social accountability approaches to help improve the performance of health systems in developing countries.^2^^–^^4^ Accountability includes the obligation of individuals or agencies to provide information about and/or justification for their actions to other actors, along with the imposition of sanctions for failure to comply and/or to engage in appropriate action.^5^^–^^9^ One of the key interventions to ensure the sustainability of gains in the health sector is to promote community ownership and engagement in health care. The community scorecard (CSC) process allows communities and service providers to engage in a dialogue on the delivery of services under a government program or a project, often in rural areas. To this end, CSCs have been most commonly used in health sectors in different African countries as a way for communities and service providers to work together on the planning and monitoring of specific health services and to jointly gear efforts toward improving service equity, quality, and access to services in resource-limited settings.^4^^,^^10^^–^^15^

Since 2006, the Ethiopian Social Accountability Program led by the Ministry of Finance and Economic Commission has been implementing CSCs in 223 districts (woredas)—third-level administrative divisions of Ethiopia covering 60,000–100,000 people—to improve the citizens' participation in public services including education, health, water and sanitation, agriculture, and rural roads.^10^ The Ethiopian Ministry of Health and regional health bureaus, along with development partners, aim to promote the involvement and engagement of community members in the planning, development, implementation, and monitoring and evaluation processes of health service delivery.^16^

**FIGURE 1:**
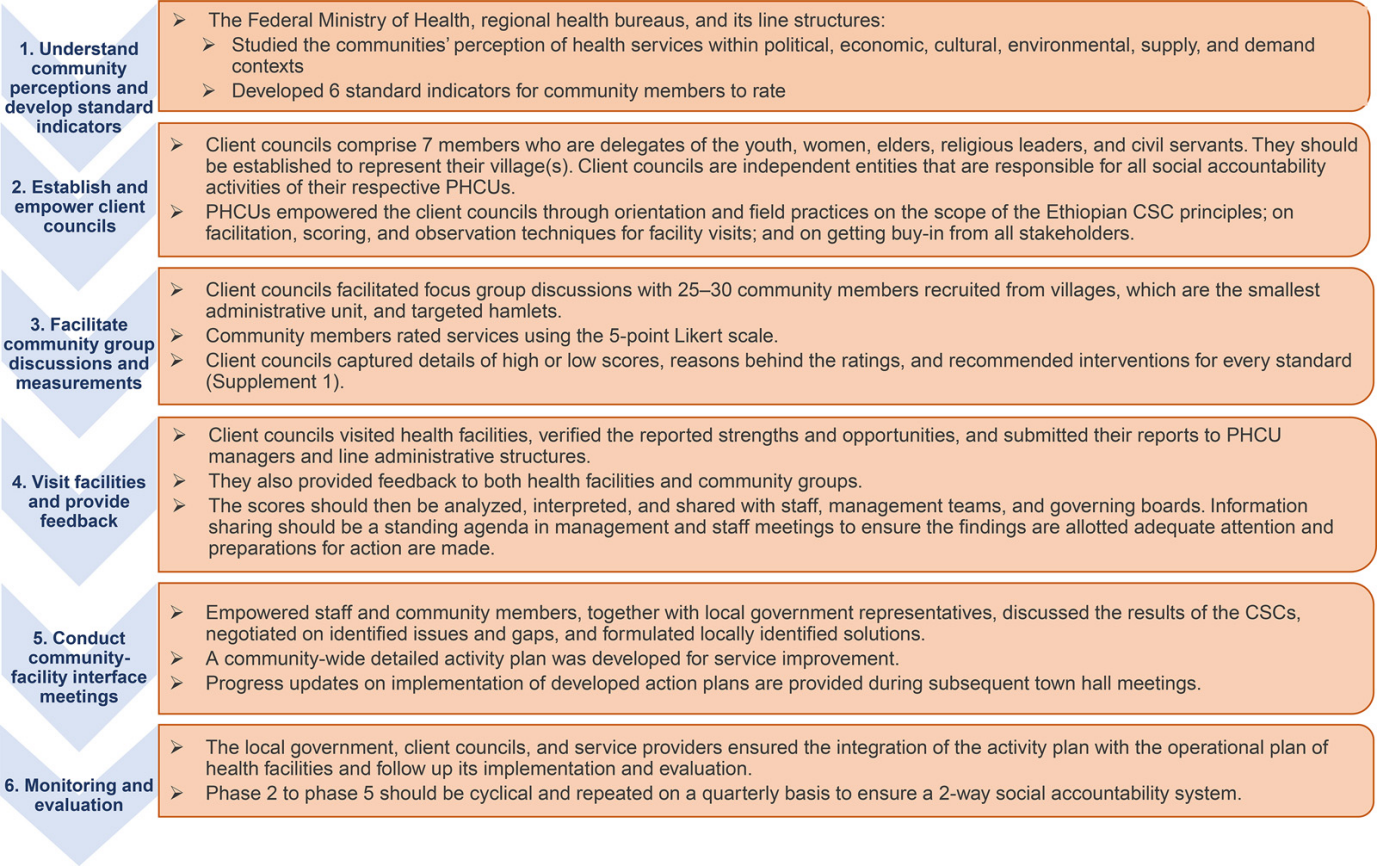
Six Phases of Implementation of a Community Scorecard Approach in Ethiopia Abbreviations: CSC, community scorecard; PHCU, primary health care unit.

During the health sector transformation plan period (2015–2020), a guide for promoting and implementing CSC approaches in the health sector was introduced. The national implementation guide dictates that community groups (i.e., client councils, health service providers, development partners, and local government officials) work together to make basic health services equitable, effective, efficient, transparent, responsive, and accountable.^17^ The guide recommends a 6-phase implementation process (Figure 1).

**FIGURE 2:**
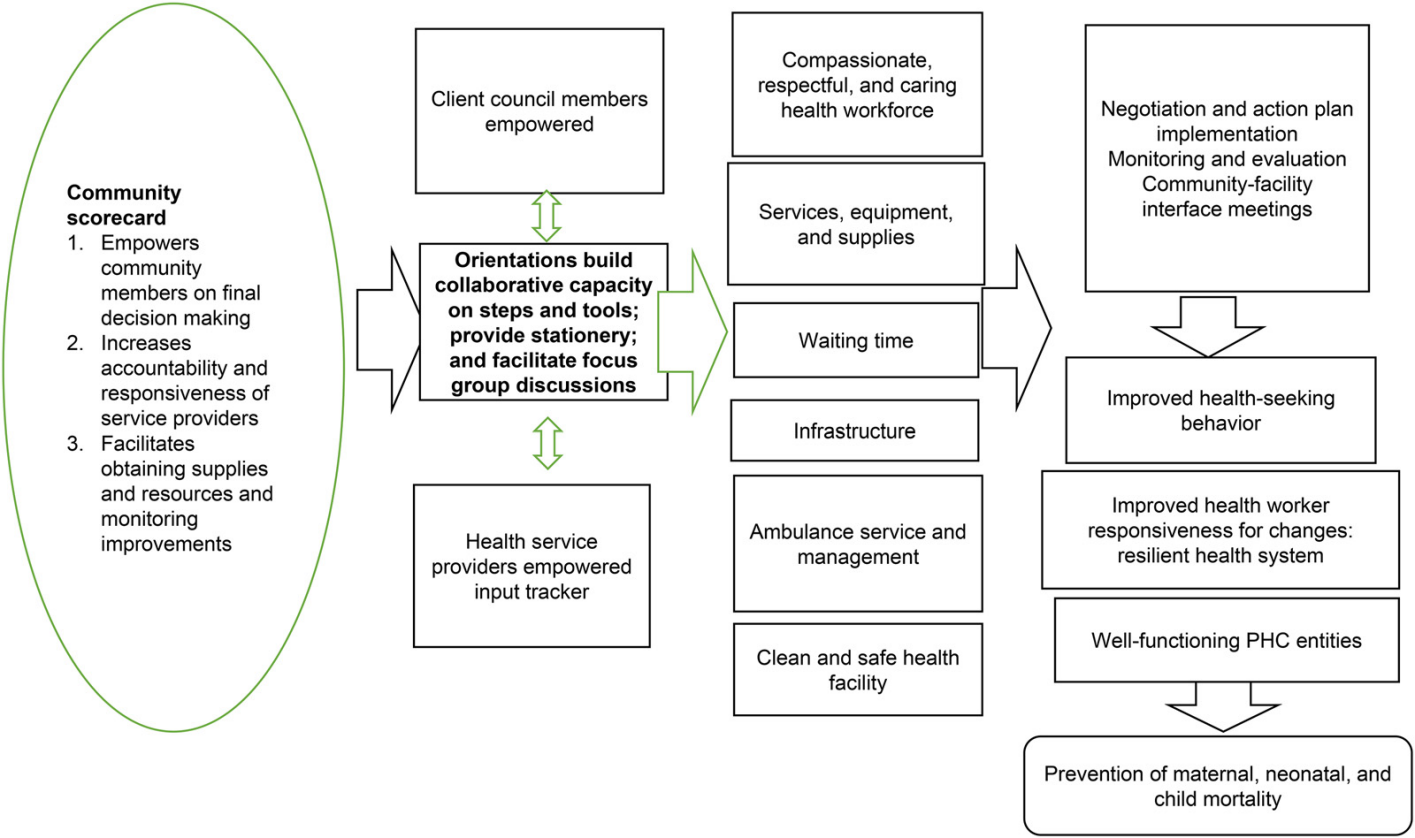
Illustration of the Ethiopian Health Sector Community Scorecard Theory of Change Developed by USAID Transform: Primary Health Care and the Ethiopian Ministry of Health Abbreviations: PHC, primary health care; USAID, United States Agency for International Development.

The exercise of developing a national strategy led to the development of a theory of change. The goal of implementing CSCs is to contribute to the prevention of maternal and child deaths.^16^^–^^18^ The long-term outcomes strategize increasing utilization of quality maternal and child health services under well-functioning primary health care entities.^18^^,^^19^ Figure 2 illustrates the preconditions and assumptions of the CSC theory of change led by the Ethiopian health sector.^20^ The preconditions are expected to enable the achievement of higher scores on (1) compassionate, respectful, and caring health workforce; (2) patient waiting time; (3) availability of services, biomedical equipment, and pharmaceutical supplies; (4) health facility infrastructure; (5) ambulance service and management; and (6) clean and safe health facility. Higher-level community scores on these standards lead to enhanced positive results on immediate expected outcomes, which include improving knowledge of entitlements and/or rights to increase health-seeking behavior of community members and subsequently improve their equitable access to quality health services. Similarly, it improves the responsiveness of health service providers or duty bearers for varied changes within the community, making the system resilient.

The U.S. Agency for International Development (USAID) Transform: Primary Health Care is a bilateral project, implemented by a consortium of international and local development partners, in collaboration with communities and the public health sector in 4 regional states in Ethiopia.^18^ The project has been providing technical, financial, and other resource support on leadership, management, and governance; health information systems; health care financing; maternal, neonatal, and child health; immunization; reproductive health; adolescent health and development; and performance and quality improvement initiatives to the public health sector to prevent avoidable maternal and child deaths.^18^

The primary health care system of Ethiopia is the foundation of the country's 3-tier health system. It is composed of a district hospital and 3 or 4 health centers, each overseeing about 5 health posts. A PHCU includes 1 health center and 5 satellite health posts, serving an average of 25,000 people.^16^ Between October 2018 and September 2019, the project initiated the CSC approach and has scaled up the initiative to 159 PHCUs in 31 districts within the Amhara and Southern, Nations, Nationalities and Peoples' (SNNP) regional states. The project has been providing technical support through building the capacity of community members and health care providers, enhancing the use of data for evidence-based decision making, adopting and printing job aids, and ensuring the responsiveness of the health system to community needs.^18^ The CSC process is considered by the Ministry of Health to be a set of evidence-based tools and resources that will promote performance and quality improvement and has been endorsed for implementation by all PHCUs.

To date, to our knowledge, no studies have assessed the effects of CSCs on health system performance in Ethiopia in terms of service utilization using longitudinal data. Despite the shortage of rigorous evidence on the inputs, process, and outcomes of implementing CSCs in Ethiopia, wide use of CSCs by the public health sector for performance improvement has continued based on the global evidence. Therefore, the results of this longitudinal pre-post interventional study present the effect of CSCs on maternal and child service performance in 2 administrative zones of Ethiopia.

To our knowledge, no studies have assessed the effects of CSCs on health system performance in Ethiopia in terms of service utilization using longitudinal data.

## METHODS

### Study Setting

This study was conducted in South Wollo and Kembata Tembaro administrative zones of Amhara and SNNP regional states, respectively. The USAID Transform: Primary Health Care project provides technical, financial, and other resource support to 91 districts in Amhara and 84 in SNNP, as well as to districts in other regional states.^20^ The project supported 31 districts and 159 PHCUs to start and implement the CSC intervention for over 12 months as a social accountability tool for performance management.^21^

Ethiopia has a 3-tier health care delivery system. Level 1 is the district (woreda) level, composed of primary hospitals that cover 60,000–100,000 people, health centers serving 15,000–25,000 people, and their satellite health posts covering 3,000–5,000 people, connected to each other through a referral system. The primary hospitals, health centers, and health posts form PHCUs. Districts are subdivided into kebeles (villages), the lowest administrative units. Level 2 includes general hospitals covering 1–1.5 million people. Level 3 includes specialized hospitals covering 3.5–5 million people.^16^

In the last 3 decades, the country has expanded access to primary health care through 17,187 health posts; 7,245 private health facilities; 3,724 health centers; and 266 hospitals. In addition, more than 151,053 health professionals are serving communities.^20^

### Implementation of the CSC Intervention

To institutionalize accountability and transparency as a tool for performance management in the health system in both Amhara and SNNP regional states of Ethiopia, 632 participants attended the CSC training of trainers, including 155 from district health offices and 477 from PHCUs. A 3-day classroom theoretical orientation and practical sessions were facilitated in July 2018 at the respective capital cities of the regional states. The CSC orientations were given to 4,053 client councils within the targeted 159 health centers in South Wollo and Kembata Tembaro administrative zones in August 2018.^21^ The client councils were informed of the 6 standards of the CSC: (1) compassionate, respectful, and caring health workforce; (2) patient waiting time; (3) availability of services, biomedical equipment, and pharmaceutical supplies; (4) health facility infrastructure; (5) ambulance service and management; and (6) clean and safe health facility, citizens' rights, service providers' duties, facilitation techniques, counting and organizing scores, observation skills, verification tools, report submission, and provision of feedback. In addition, every 3 months, the client councils were actively engaged in facility-community interface meetings, presented the results of the CSCs and feedback of community members, addressed issues raised by town hall meeting participants, and closely monitored the implementation of developed action plans. The project supported all client councils with 1-page job aids, reporting forms, and a minute book.

From September 2018 to December 2019, USAID Transform: Primary Health Care provided technical, financial, and other resource support to its targeted districts. Some forms of this support were providing team-based strategic problem-solving trainings for health care providers with the formation of performance improvement and quality improvement projects, enhancing the capacity of performance management team members through offering the use of data for decision-making trainings, providing performance improvement subgrant funding for primary health care entities, and organizing and facilitating community-facility interface meetings.

In addition, the project's staff and experts from zone health departments provided follow-up visits and on-site coaching for all PHCUs and district health offices on a quarterly basis. During facility visits, the coaching team facilitated the exploration of CSC measurements with feedback and gave opportunities for PHCU management staff to systematically analyze the root causes of any issues, propose prioritized solutions, and develop doable action plans. In addition, the coaches revised the concepts of social accountability, provided feedback on performance, and supported the client councils and health care providers. The coaches then submitted a copy of agreed measurement reports, identified gaps, and developed action plans with district health offices and to zone health departments. Baseline data were extracted from the period of October 10–20, 2018. At 6 and 12 months after the intervention periods, secondary data were collected during April 10–20, 2019, as midterm assessments, and during October 10–22, 2019, as endline measurements by the data collection teams.^21^

### Study Design

For this study, the investigators used a longitudinal pre-post interventional study design.^22^ The necessary data were collected from October 2018 to September 2019. We used quantitative methods to measure the effects of CSC implementation on health-seeking behavior, health service utilization, and responsiveness of health care providers.

### Sample Size

The study participants were identified from the Amhara and SNNP regional states in Ethiopia. Two administrative zones where the project had provided technical and other resource support for 12 months or more were purposively selected. All 31 districts (woredas) and 159 PHCUs were included in selected zone administrations. Documents and records from each primary health care entity were reviewed at the 3 points of the assessment.

### Data Collection Procedures

The required routine health management information system data on CSC and on maternal and child health services were extracted from 159 PHCUs' RHIMS database. To ensure data completeness, accuracy, consistency, and reliability, 8 data managers and 2 supervisors were trained for 3 days. The training covered the objective of the pre-post interventional study, ethical issues, quantitative data extraction methods, and piloting all tools and ethical principles. During the real data collection, all investigators actively monitored completeness and consistency of data on a daily basis. Data were extracted using structured and pretested forms.

**Dependent variables:** The average measures, from 0% to 100% on 10 key performance indicators (KPIs) were reviewed and collated: (1) contraceptive acceptance rate, (2) syphilis screening among antenatal care (ANC) clients, (3) skilled delivery services coverage, (4) postnatal care coverage, (5) full immunization coverage, (6) under 2 years growth monitoring coverage, (7) proportion of available essential or tracer drugs, (8) proportion of clean and safe health facility standards met, (9) proportion of available laboratory and diagnostic services, and (10) patient flow and service organization (Table 1).^18^^,^^21^ The effects of CSC implementation on the health system's performance were measured at baseline, midterm (after 6 months), and endline (after 12 months).

**TABLE 1.** Description of Key Performance Indicators and Community Scorecard Scoring Standards to Assess Effects of Implementing a Social Accountability Approach on Improving Health System Performance in Maternal and Child Health Services in 2 Regional States in Ethiopia^a^

**I. Key Performance Indicators**	
Contraceptive acceptance rate	
Number of women of reproductive age who use family planning	×100%
Number of women eligible for modern family planning methods	
Syphilis screening among pregnant women attending antenatal care (ANC)	
Number of pregnant women tested for syphilis	×100%
Total number of pregnant mothers attended at least 1 ANC visit	
Skilled delivery service coverage	
The number of births attended by skilled health personnel	×100%
Total number of expected deliveries	
Postnatal care service coverage	
Number of postnatal visits within 7 days of delivery	×100%
Total number of expected deliveries	
Fully immunization coverage	
Number of children who received all vaccine doses before first birthday	×100%
Total number of surviving infants	
Under 2 years growth monitoring service coverage	
Number of children less than 2 year weighed during growth monitoring session	×100%
Total estimated children under 2 years	
Availability of essential (tracer drugs)	
Sum of tracer drugs × months available in the time period	×100%
Sum tracer drugs × sum total number of months in time period	
Clean and safe facility	
Number of clean and safe facility minimum standards met	×100%
Total number of clean and safe facility minimum standards (met and unmet)	
Availability of laboratory and diagnostic services	
Number of laboratory services minimum standards met	×100%
Total number of laboratory services minimum standards (met and unmet)	
Patient flow and service organization	
Number of patient flow and service organization minimum standards met	×100%
Total number of patient flow and service organization minimum standards (met and unmet)	
**II. Community Scorecard Facilitation and Scoring**	
**Indicator 1: Compassionate, respectful, and caring health workforce**	
Consider patients as human beings, and provide person-centered care with empathy; effective communication with health care teams and in interactions with patients; and respect for and facilitation of patients' and families' participation in decisions and care	
**Indictor 2: Patient waiting time for health care services**	
Waiting time refers to the time from the patient's arrival at the health facility to the time the patient receives services	
**Indicator 3: Availability of services, biomedical equipment, and pharmaceutical supplies**	
Availability of services, biomedical equipment, and pharmaceutical supplies	
**Indicator 4: Health facility infrastructure**	
Health facility has adequate infrastructure, such as appropriate building, electricity, and water, and the infrastructure is functional for patient care.	
**Indicator 5: Ambulance service and management**	
Ambulance service is readily available whenever it is required, and transparent and appropriate ambulance car service management is in place.	
**Indicator 6: Clean and safe health facility**	
Health facility compound is clean, green, and pleasing; clinical service areas, such as the outpatient rooms, inpatient beds, and laboratory service area, are safe, hygienic, and odor free; and waste disposal mechanism managed without risk to the health workers, patients, and community members.	

a Ten selected maternal, neonatal, and child health indicators were rated from 0.0% to 100.0% and the average scores were extracted. Similarly, the community scorecards were measured against 6 minimum standards developed by the Ministry of Health, regional health bureaus, and development partners. The overall score was rated from 0.0% to 100.0% and extracted from the database.

**Independent variables:** The data extraction forms dedicated to capture information on CSCs were adopted from the nationally endorsed CSC reporting tools. The forms captured the summary of the following 6 standards: (1) compassionate, respectful, and caring health workforce; (2) patient waiting time; (3) availability of services, biomedical equipment, and pharmaceutical supplies; (4) health facility infrastructure; (5) ambulance service and management; and (6) clean and safe health facility, reported as an average score from a 5-point Likert scale measurement (Supplement 1), that is, 5=very good to 1=very low. The overall summary of CSCs was reported as a percentage from 0% to 100.0% on 3 occasions^17^ (Table 1). The data were collated using a Microsoft Excel sheet and exported to Statistical Program for Social Science (SPSS IBM V 20) software for analysis. To ensure consistency and reliability, the data were double entered by experienced data encoders.

### Data Analysis

The PHCU scores of CSCs and KPIs were reviewed for completeness and consistency. Quantitative data analysis methods were used, which included descriptive statistic frequencies, mean, median, interquartile ranges, and standard deviations. To determine the presence of a linear relationship between baseline, midterm, and endline scores, a Pearson Product-Moment Correlation technique was employed. After checking the assumption of the nonparametric test, we used the Friedman^23^ test, which includes (1) a single group measured on 3 or more different occasions, (2) a group that is a random sample from population, (3) use of a continuous level of dependent variables, and (4) samples that do not need to be normally distributed. Post hoc analysis with the Wilcoxon signed-rank tests was conducted with a Bonferroni correction applied and a statistical test result with a *P*-value of <.017, indicating presence of a significant difference between CSCs and KPIs at baseline, midterm, and endline measurements.

### Ethical Considerations

The ethical clearances of this study were granted by the JSI Institutional Review Board (IRB), and the Amhara Public Health Institute and SNNP Regional State Health Bureaus' IRBs.

The research protocol of this pre-post interventional study was granted certification from the Amhara Public Health Institute (reference number: M/T/SH/D/03/435) and the SNNP Regional State Health Bureau Research and Ethics Review Committees (reference number NS 12/36/22). In addition, the study protocol was reviewed at the JSI Research & Training Institute, Inc. IRB, which determined that this activity was exempt from human subjects' oversight (reference number IRB no. 19-16E). To maintain the confidentiality of collected data, anonymity was maintained throughout the research process.

Permission to use data from zone health departments and PHCUs were obtained through formal written requests. Informed individual written consent was taken from each study subject. Both quantitative and qualitative data were collected in aggregate forms. All PHCUs were encouraged to use the data for evidence-based decision making and to be responsive to the demands of rights holders. The summary of CSCs and KPIs were submitted to district health offices, zone health departments, and regional health bureaus at the 3 points of the assessment. Throughout the research process, the investigators maintained national and international ethical principles.

## RESULTS

In this study, a total of 31 districts from 2 regional states of Ethiopia were enrolled. Table 2 depicts the characteristics of the study areas. Two-thirds (67.7%) of the districts and the majority (128; 80.5%) of PHCUs were located in South Wollo administrative zones of Amhara region. About 3.8 million inhabitants reside in the study area there. A total of 38,556 community representatives participated in focus group discussions and measurements.

**TABLE 2.** Characteristics of 2 Regions in Ethiopia Where the Study Assessed the Effects of Implementing a Social Accountability Approach on Improving Health System Performance in Maternal and Child Health Services

	SNNP RegionNo. (%)	Amhara RegionNo. (%)	Total
Districts	10 (32.3)	21 (67.7)	31
Primary health care units	31 (19.5)	128 (80.5)	159
Population	814,564 (21.2)	3,022,075 (78.8)	3,836,639
Number of villages (kebeles)	166 (22.6)	568 (77.4)	734
Client councils	31 (5.4)	548 (94.6)	579
Villages that implemented community scorecard	93 (14.5)	548 (85.4)	641
Client council members participated in focus group discussions	217 (5.3)	3,836 (94.7)	4,053
Community members participated in focus group discussions	2,604 (6.7)	35,952 (93.3)	38,556

Abbreviation: SNNP, Southern, Nations, Nationalities and Peoples.

### Positive Changes in Overall Average CSC and KPI Scores

Table 3 presents the summary score of CSCs and KPI values. The percentage of CSC and standard deviation (±SD) achieved at the 3 points in time were 60.8% (±12.5%), 66.3% (±10.8%), and 70.6% (±10.0%) for baseline, midterm, and endline measurements, respectively. The mean CSC measurement improvement was observed from 68.7% to 74.4% and 58.9% to 69.7% in Kembata Tembaro and South Wollo administrative zones, respectively. Availability and utilization of services were higher at 3 points in time, that is, 54.9±17.4, 61.9±15.1, and 67.6±14.6 at baseline, midterm, and endline, respectively. The mean KPI (±SD) scores of 31 health centers in Kembata Tembaro zone were 75.6% (±19.1%), 79.7% (±7.5%), and 80.9% (±8.7%) at baseline, midterm, and endline measurements, respectively. Similarly, the mean KPI scores (±SD) of 128 health centers in South Wollo zone were 49.9% (±15.1%), 57.6% (±13.1%), and 63.7% (±13.2%) at baseline, midterm, and endline measurements, respectively. The results revealed the presence of significant positive changes in syphilis screening tests among ANC clients (58.6% to 78.4%), skilled delivery attendance (40.6% to 54.9%), full immunization services (78.5% to 83.0%), and availability of essential or tracer drugs (51.3% to 69.6%) in South Wollo zone. There were also improvements in the Kembata Tembaro zone within the SNNP region, including syphilis screening tests among ANC clients (82.9% to 87.8%), skilled delivery attendance (64.7% to 66.9%), growth monitoring services (84.3% to 92.8%), and availability of essential or tracer drugs (63.2% to 76.5%).

**TABLE 3.** Key Performance Indicator and Community Scorecard Scores of the Study Assessing the Effects of a Social Accountability Approach on Improving Health System Performance in Maternal and Child Health Services in 2 Regional States in Ethiopia, October 2018 to September 2019^a^

	Baseline		Midterm		Endline	
	SNNP	Amhara	SNNP	Amhara	SNNP	Amhara
Social accountability						
Compassionate, respectful, and caring health work force	3.78±0.50	3.07±0.72	3.56±0.53	3.41±0.68	3.77±0.45	3.66±0.60
Patient waiting time	3.31±0.97	2.95±0.80	3.39±0.60	3.25±0.72	3.61±0.57	3.47±0.77
Availability of services, biomedical equipment, and pharmaceutical supplies	3.40±0.61	2.92±0.68	3.74±0.45	3.15±0.70	3.83±0.52	3.40±0.67
Health facility infrastructure	3.44±0.70	2.86±0.74	3.69±0.53	3.15±0.82	3.71±0.57	3.47±0.71
Ambulance service and management	3.11±0.55	2.57±0.81	3.29±0.51	3.06±0.81	3.58±0.48	3.14±0.80
Clean and safe health facility	3.53±0.50	3.28±0.88	3.67±0.79	3.50±0.78	3.84±0.78	3.75±0.68
Overall summary of community scorecard	68.7±6.6	58.9±12.8	71.2±5.8	65.2±11.5	74.4±6.4	69.7±10.5
	60.8%±12.5%		66.3%±10.8%		70.6%±10.0%	
Key performance indicators						
Contraceptive acceptance rate	80.7±22.0	85.2±28.4	82.7±25.0	93.4±22.0	88.9±22.3	95.1±28.7
Syphilis screening among antenatal care clients	82.9±17.7	58.6±37.3	84.2±17.5	67.4±36.5	87.8±22.6	78.4±43.1
Skilled delivery service coverage	64.7±23.4	40.6±21.5	66.3±23.3	45.0±22.0	66.9±23.5	54.9±28.0
Postnatal service coverage	76.1±23.3	52.7±25.9	88.6±18.6	56.6±24.0	84.5±23.1	67.4±31.4
Fully immunization coverage	98.2±15.2	78.5±25.9	94.3±7.8	76.8±18.7	97.7±14.7	83.0±20.4
Growth monitoring coverage	84.3±29.6	13.9±7.1	87.9±25.4	14.5±8.5	92.8±18.8	17.0±10.9
Proportion of available essential or tracer drugs	63.2±16.7	51.3±32.0	72.5±14.3	63.3±24.8	76.5±13.5	69.6±20.1
Proportion of available diagnostic services	74.2±20.4	37.0±37.7	83.2±17.0	50.4±35.8	86.5±12.9	51.0±36.2
Proportion of clean and safe health facility standards met	77.0±18.2	49.4±33.8	80.9±15.1	63.1±26.1	85.0±11.0	70.4±21.8
Waiting time/ patient flow/responsiveness health center–health post linkage	54.8±25.1	31.7±30.1	56.9±26.4	45.1±30.5	69.3±27.1	50.0±29.2
Overall key performance indicators summary score	75.6±19.1	49.9±15.1	79.7±7.5	57.6±13.1	83.6±7.1	63.7±13.2
	75.6%±9.1%		79.7%±7.5%		80.9%±8.7%	

Abbreviation: SNNP, Southern, Nations, Nationalities and Peoples.

a Average community scorecard and key performance indicators with standard deviation.

### Difference in CSC Values Over Time

At baseline, the mean score of CSCs was 60.8; at midterm, it was 66.3; and at endline, it was 70.6. A statistically significant difference was found in CSC ratings at baseline, midterm, and endline measurements, χ^2^(2)=252.642, *P*=.000. Post hoc analysis using the Wilcoxon signed-rank tests was conducted with a Bonferroni correction applied, resulting in a significance level set at *P*<.017. Median (interquartile range) CSC measurements at baseline, midterm, and endline were 60.4 (52.9–70.3), 66.9 (58.3–73.9), and 71.0 (61.1–77.6), respectively. There were statistically significant positive differences between CSC at midterm and baseline (*Z*=−9.049, *P*=.00); endline and baseline (*Z*=−10.235, *P*=.000), and endline and midterm (*Z*=−9.667, *P*=.000). Therefore, it can be concluded that long-term community engagement elicits a significant rise in perceived CSC measurement values.

### Difference in KPIs Over Time

At baseline, the mean score of KPIs was 54.94; at midterm, it rose to 61.93; and at endline, it increased even further to 67.61. A statistically significant difference was found in KPIs at baseline, midterm and endline measurements, χ^2^(2)=267.142, *p*=0.001. Post hoc analysis with Wilcoxon signed-rank tests was conducted with a Bonferroni correction applied, resulting in a significance level set at *P*<.017. Median (interquartile range) KPI measurements at baseline, midterm, and endline were 53.6 (42.0–68.5), 60.1 (51.6–74.5), and 66.3 (56.7–80.0), respectively. There were statistically significant positive differences between KPI at midterm and baseline (*Z*=–10.203, *P*=.001), endline and baseline (*Z*=−10.889, *P*=.001), and endline and midterm (*Z*=−10.026, *P*=.000). Therefore, it can be concluded that long-term community engagement brings a significant increase in KPI measurement values.

Table 4 shows that the summary result of each CSC and average KPIs have a positive correlation, as CSC at midterm and average KPI at endline had a moderate degree relationship (r>0.377). The highest correlation was seen between KPI at midterm and endline (r>0.924), which was followed by KPI at baseline and midterm (r>0.882). These findings also indicate a statistically significant correlation between CSC and KPIs.

**TABLE 4.** Correlation Summary Analysis of Each Community Scorecard With Average Key Performance Indicators Score of the Study Assessing the Effects of a Social Accountability Approach on Improving Health System Performance in Maternal and Child Health Services in 2 Regional States in Ethiopia, October 2018 to September 2019

		CSC Baseline	CSC Midterm	CSC Endline	KPI Baseline	KPI Midterm	KPI Endline
CSC baseline	Pearson Correlation	1					
	Sig. (2-tailed)						
	N	159					
CSC midterm	Pearson Correlation	.718^a^	1				
	Sig. (2-tailed)	.000					
	N	159	159				
CSC endline	Pearson Correlation	.662^a^	.874^a^	1			
	Sig. (2-tailed)	.000	.000				
	N	159	159	159			
KPI baseline	Pearson Correlation	.504^a^	.489^a^	.533^a^	1		
	Sig. (2-tailed)	.000	.000	.000			
	N	159	159	159	159		
KPI midterm	Pearson Correlation	.448^a^	.412^a^	.446^a^	.882^a^	1	
	Sig. (2-tailed)	.000	.000	.000	.000		
	N	159	159	159	159	159	
KPI endline	Pearson Correlation	.398^a^	.377^a^	.429^a^	.815^a^	.924^a^	1
	Sig. (2-tailed)	.000	.000	.000	.000	.000	
	N	159	159	159	159	159	159

Abbreviations: CSC, community scorecard; KPI, key performance indicator.

a Correlation is significant at the 0.01 level (2-tailed). Perfect: If the value is near ±1, then it said to be a perfect correlation: as one variable increases, the other variable tends to also increase (if positive) or decrease (if negative). High degree: If the coefficient value lies between ±0.50 and ±1, then it is said to be a strong correlation. Moderate degree: If the value lies between ±0.30 and ±0.49, then it is said to be a medium correlation. Low degree: When the value lies below + 0.29, then it is said to be a small correlation. No correlation: When the value is zero.

## DISCUSSION

This longitudinal, pre-post CSC interventional study in 31 districts and 159 PHCUs documented the establishment of 579 client councils and the participation of 38,556 community members. The support of the project, which included capacity building of health workers and community members, as well as provision of job aids, reporting forms, and performance improvement subgrants, helped the PHCUs to increase their performance. The baseline, midterm, and endline data showed statistically significant ongoing increases. The results revealed positive correlations between the CSC average values and the average KPI achievements. Therefore, it can be concluded that building the capacity of community members and health workers on the implementation of social accountability interventions can help to improve service availability and utilization of primary health care services.

The support of the project, including health worker capacity building, job aids, and reporting forms, helped the PHCUs to increase their performance.

The measurement results of CSCs were positively correlated and had statistically significant higher differences, which may have occurred due to the responsiveness of the health system to the needs and demands of community members in terms of health worker behavior, availability of services, and improvements in supplies, infrastructure, and waiting time. This finding was in line with an article by Ho et al.^24^ on CSC implementation in the DRC, which showed improved patient and health provider relationships as well as improved quality of and access to primary health care services.^24^ Tabish^25^ and Herrera et al.^26^ confirmed that changing governance arrangements had an effect on health outcomes. Similarly, in Afghanistan, Edward et al.^27^ attested that the implementation of CSCs helped to enhance governance and health system accountability for people-centered health care.

**FIGURE 3:**
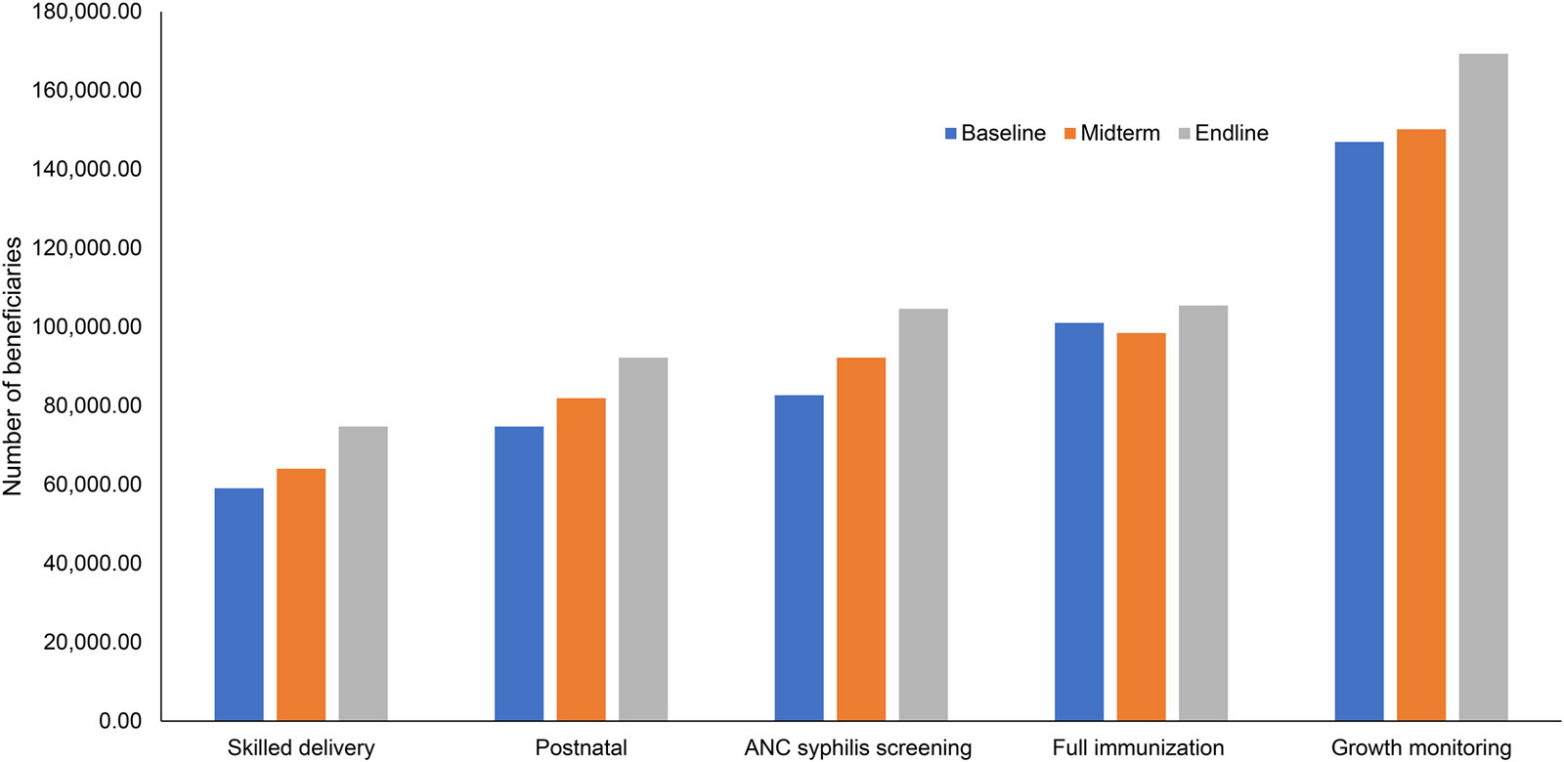
Increased Utilization of Maternal and Child Health Services in Ethiopia, October 2018 to September 2019 Abbreviation: ANC, antenatal care.

Maternal and child health service utilization increased, specifically for skilled delivery (from 45.3% to 57.3%), postnatal care services (from 57.3% to 70.7.0%), syphilis screening (from 63.4% to 80.2%), full immunization for infants under 1 year (from 82.3% to 85.9%), and growth monitoring for children under 3 years (from 27.0% to 31.8%) (Figure 3). These results could have been achieved through negotiations between community members and health workers in which detailed action plans were developed, implemented, and evaluated (Supplement 2). This finding was in line with Gullo et al.,^14^ Schaaf et al.,^4^ and Gullo et al.,^15^ who found that service utilization was significantly higher among CSC intervention groups than among nonintervention community members. Similarly, Blake et al.^28^ attested that after a 1-year implementation of scorecards in 37 health facilities in Ghana, there was a significant improvement on maternal and newborn health services through better access to essential drugs, improved infrastructures, and availed essential equipment.

The implementation of CSCs at the PHCU level was aligned with the performance measurement and enhancing accountability, transparency, and engagement of public and civil societies and duty holders (citizens) to achieve the strategic objectives of the Ethiopian Health Sector Transformation Plan (HSTP 2015-2020) of the Ethiopian government.^16^^,^^29^ This finding was in line with Argaw et al.,^19^ who reported that measurements against minimum standards and developing improvement plans helped to improve primary health system performance. In addition, this social accountability tool is implemented by creating pools of trainers, orienting health care providers and community members on the principles and guiding steps, and providing job aids. However, to improve the performance of the health system, costs associated with close follow-up on action plans by the next higher-level institution in the system and performance improvement funds should be a part of the CSC intervention.

This longitudinal pre-post interventional study revealed that the implemented CSC intervention, which is a nationally recommended citizen measurement, engagement, and accountability tool, helps health workers and community members to contribute to the improvement of the performance of the health system.

### Limitations

This study has some limitations. The main limitation is related to the pre-post study design; unlike a randomized study design, it is difficult to conclude the causal association between the CSC intervention and the KPI improvements with a pre-post design. The other limitation of this study is the lack of literature on government-led large-scale implementation of CSC. Hence, this study uses results from projects implemented by civic society organizations in limited areas. As the study targeted 2 zone administrations, before generalizing the findings, the context should be noted.

## CONCLUSIONS AND RECOMMENDATIONS

Based on this study's results, implementing CSCs as a tool to enhance accountability, transparency, and community engagement could help to contribute to improvements in the performance of the health system regarding maternal and child health services. Ensuring continuity of the implementation of the CSC intervention by orienting client councils on the principles of CSCs, providing reporting forms and job aids, and providing subgrants for PHCUs is recommended. In addition, a qualitative study to document the process of CSC implementation and experiences of community representatives is recommended.

## Supplementary Material

20-00114-Argaw-Supplement2.pdf

20-00114-Argaw-Supplement1.pdf

## Notes

**Peer Reviewed**
