# Supplementary material for: Implementing a Social Accountability Approach for Maternal, Neonatal, and Child Health Service Performances in Ethiopia: A Pre-Post Study Design
Source: Glob Health Sci Pract. 2021 Mar 31;9(1):123–35. doi: 10.9745/GHSP-D-20-00114 (PMC8087434; doi:10.9745/GHSP-D-20-00114)
Supplement: 20-00114-Argaw-Supplement2.pdf [file 20-00114-Argaw-Supplement2.pdf]

Supplement to: Argaw MD, Fekadu BD, Mamo E, et al. Implementing a social accountability approach for maternal, neonatal, and child health service performances in Ethiopia: a pre-post study design. *Glob Health Sci Pract*. 2021;9(1). https://doi.org/10.9745/GHSP-D-20-00114

Supplement 2: Action Plan based on community and health workers negotiation, Gimba Health Center, Jan 2019

Ser. No.	CSC indicators	What can we do to make things better?	Who will do this?	By when
1	Compassionate, Respectful and Caring workforce	• Train one staff on CRC who can train other staff<br>• Provide CRC training to all staff<br>• Establish CRC self-assessment tool	Health center director<br>All staff & Health center director	30 Oct 2018-<br>31 Dec 2018
2	Waiting time for health services	• Alphabetize patient card and implement MPI box properly<br>• Establish Triage services<br>• Post services, time and associated cost to users	HIT & all staff<br>Health center director<br>Lab head & other staff	30 Oct 2018 -<br>31 Dec 2018
3	Availability of drugs, supplies and diagnostic services	• Address drug and supply stock out (procurement)<br>• Budget request<br>• Facilitate loan from CBHI scheme<br>• Resume outreach services	Pharma head<br>& DTC committee<br>Health center director	30 Oct 2018 -<br>31 Dec 2018
4	Infrastructure of the health facility	• Purchase electric generator<br>• Establish functional maternal waiting home	Health center head (board)	30 Oct 2018 -<br>31 Dec 2018
5	Availability and management of ambulance services	• Provide 24-hour ambulance services (hiring additional drivers)	Health Center Head (Board)	30 Oct 2018 -<br>31 Dec 2018
6	Clean and Safe Health Facility	• Establish CASH committee and establish a cleaning campaign, purchase IP materials<br>• Work for aesthetic	CASH focal person<br>Other staff<br>Youth club members	30 Oct 2018 -<br>31 Dec 2018

Supplement to: Argaw MD, Fekadu BD, Mamo E, et al. Implementing a social accountability approach for maternal, neonatal, and child health service performances in Ethiopia: a pre-post study design. *Glob Health Sci Pract*. 2021;9(1). https://doi.org/10.9745/GHSP-D-20-00114
