# Supplementary material for: Implementing a Social Accountability Approach for Maternal, Neonatal, and Child Health Service Performances in Ethiopia: A Pre-Post Study Design
Source: Glob Health Sci Pract. 2021 Mar 31;9(1):123–35. doi: 10.9745/GHSP-D-20-00114 (PMC8087434; doi:10.9745/GHSP-D-20-00114)
Supplement: 20-00114-Argaw-Supplement1.pdf [file 20-00114-Argaw-Supplement1.pdf]

Supplement to: Argaw MD, Fekadu BD, Mamo E, et al. Implementing a social accountability approach for maternal, neonatal, and child health service performances in Ethiopia: a pre-post study design. *Glob Health Sci Pract*. 2021;9(1). https://doi.org/10.9745/GHSP-D-20-00114

Supplement 1: Community score card indicators matrix

Proposed CSC Indicators	Measures	Criteria for rating (data elements)				
		1=Very low	2= low	3=ok	4=Good	5=Very good
1. Caring, respectful, and compassionate care.	a) Shows respect to patients<br>b) Shows compassion<br>c) Receives patients well<br>d) Has passion for the patient service	Fulfils none of the measures	Fulfils one of the measures	Fulfils two of the measures	Fulfils three of the measures	Fulfils all of the measures
2. Waiting time for provision of health care services	a) Fast service<br>b) Efficient service	Very slow and inefficient service	Slow service	Average service	Fast service	Very fast and efficient service
3. Availability of medicines, diagnostic services and medical supplies.	a) Availability in amount and kind<br>b) Availability in time	Very dire unavailability all the time	Frequent unavailability of most	Partly available	Available with some interruptions	Available all the time
4. Infrastructure of health facilities	a) Availability of water, electricity, road, buildings.<br>b) Regularity of availability	Severe unavailability of infrastructure resulting in interruption of services for days	Unavailability of infrastructure resulting in inefficient services	Partially available	Sufficient availability of infrastructure	All infrastructure needs are fulfilled
5. Availability and management of ambulance services	a) Utilization of ambulance services<br>b) Satisfaction with ambulance services	Service is not available or very inefficient with management problems	Does not meet the community's needs most of the time	Meets the community's needs some of the time	Meets the community's needs most of the time	Sufficiently meets the community's needs
6. Cleanliness and sanitation of healthcare facility	a) Clean and comfortable area for healthcare service provision<br>b) Patient safety	Has sever cleanliness, comfort and safety problems	Not clean, comfortable or safe for patients	Only partially clean, comfortable, and safe for patients	Sufficiently clean, comfortable and safe for patients	Very clean, comfortable and safe for patients
